# Comparative Transcriptomic Analysis of Chlorophyll Metabolism in Broccoli Under Preharvest 1-MCP Application Versus Pre-Cooling Combined with Cold Chain Storage

**DOI:** 10.3390/foods15101688

**Published:** 2026-05-12

**Authors:** Li Zhang, Tengfei Liu, Yingying Zhu, Libin Wang, Xiaoyu Xie, Li Jiang

**Affiliations:** 1 Food & Medicine Homology Big Health Innovation Consortium, Suzhou Polytechnic University, Suzhou 215104, China; zhangli_szd@163.com (L.Z.); 01412@jssvc.edu.cn (Y.Z.); 2 Jiangsu Taihu Area Institute of Agricultural Sciences, Suzhou 215106, China; liutengfei@jaas.ac.cn; 3 College of Food Science and Technology, Nanjing Agricultural University, Nanjing 210095, China; lbwang88@126.com (L.W.); nau_xxiaoyu@163.com (X.X.)

**Keywords:** broccoli, pre-harvest 1-MCP, pre-cooling, cold storage, transcriptome analysis

## Abstract

Broccoli (*Brassica oleracea* var. *italica*) is highly nutritious, rich in vitamin C, glucosinolates, and minerals. However, its high postharvest respiratory rate leads to rapid quality deterioration, particularly chlorophyll degradation and yellowing under ambient conditions. In China, the lack of timely pre-cooling facilities exacerbates postharvest losses. Therefore, developing safe, effective and low-cost preservation methods for broccoli during transportation is of great practical importance. In this study, RNA sequencing was employed to analyze the effects of preharvest 1-methylcyclopropene (1-MCP) and postharvest pre-cooling combined with cold treatments on gene expression in broccoli. Transcriptome analysis revealed that both treatments significantly upregulated or maintained key genes involved in chlorophyll biosynthesis (e.g., Glutamyl-tRNA reductase (GluTR), porphobilinogen deaminase (PBGD), magnesium chelatase (MgCh)) and downregulated chlorophyll degradation-related genes (e.g., Chlorophyllase (CLH), pheophytinase (PPH), pheophorbide a oxygenase (PaO)), resulting in enhanced chlorophyll retention. Furthermore, chlorophyllide a oxygenase (CAO) was upregulated, while chlorophyll b reductase (CBR) was downregulated, suggesting modulation of the chlorophyll cycle. These findings elucidate the molecular mechanisms by which 1-MCP and pre-cooling combined with cold regulate chlorophyll metabolism, providing new insights into the gene regulatory network underlying the postharvest quality maintenance in broccoli.

## 1. Introduction

Broccoli (*Brassica oleracea* var. *italica*), an annual or biennial herbaceous plant of the *Brassicaceae* family, is a major vegetable crop in China, prized for its edible inflorescences rich in vitamins, antioxidants, and dietary fiber [1,2]. However, its postharvest marketability is often severely compromised by rapid senescence, which is characterized by floret yellowing driven by intense metabolic activity, including heightened respiration and ethylene production [3]. To extend shelf life, various postharvest strategies have been adopted. These encompass physical methods such as cold storage, heat shock, and irradiation [4,5,6], as well as chemical treatments, including ethylene antagonists (e.g., 1-methylcyclopropene [1-MCP]), plant growth regulators, amino acids, and hormone-like substances such as melatonin and jasmonic acid [7,8,9,10,11,12].

Among these, cold storage combined with rapid pre-cooling is one of the most effective methods for maintaining broccoli quality [13]. However, its timely implementation remains a logistical challenge for many small-scale farmers and distributors in China. Our field investigations indicate that the transportation of broccoli from harvest to the wholesaler’s cold storage typically lasts 18 to 24 h. During this critical period, most growers and traders lack the capacity to perform timely pre-cooling. Consequently, delays in the cold chain frequently result in significant postharvest losses before the produce reaches the consumer. This highlights an urgent need for the development of practical, cost-effective, and safe preservation techniques that are operationally feasible within the local supply chain, either to supplement or to bridge gaps in the cold chain.

As a safe and potent ethylene action inhibitor, 1-MCP has been widely used in postharvest broccoli preservation [14,15,16,17,18]. Recently, preharvest foliar application of 1-MCP has emerged as a logistically advantageous alternative, simplifying operations and reducing postharvest handling. Preharvest application of 1-MCP has emerged as a novel strategy that offers a mechanistic shift beyond the traditional postharvest usage. While postharvest treatments primarily act as a temporary ethylene receptor antagonist to delay ripening, preharvest sprays have been shown to induce long-lasting physiological changes. Notably, preharvest 1-MCP can modulate the expression of key ripening genes such as MdACS and MdACO, and influence wax biosynthesis pathways, thereby preserving fruit integrity and reducing physiological disorders like superficial scald and fruit drop [19]. This approach has shown efficacy in fruits such as apples [20,21,22,23], sweet cherries [24], and pears [25], as well as in floriculture (e.g., lilies [26] and roses [27]), though its application in broccoli remains limited. Crucially, the molecular mechanisms through which preharvest 1-MCP treatment delays senescence—especially in combination with subsequent cold storage practices—are largely unknown.

Chlorophyll metabolism is a critical determinant of postharvest quality in broccoli. Recent advances have elucidated multiple layers of regulation governing chlorophyll synthesis and degradation. Recent studies have significantly advanced our understanding of chlorophyll metabolism in broccoli (*Brassica oleracea* L. var. *italica*). Central to this progress is the elucidation of the genetic and enzymatic networks that govern chlorophyll biosynthesis and degradation. For instance, the degradation of chlorophyll, a hallmark of broccoli senescence, is primarily regulated by key catabolic genes such as BoPPH, BoPAO, BoNYC1, and BoCLH2, whose expression levels directly correlate with the rate of leaf yellowing [28]. Similarly, the application of slightly acidic electrolyzed water (SAEW) or calcium chloride-acidified electrolyzed water has been demonstrated to preserve color, reduce oxidative damage, and slow chlorophyll breakdown in broccoli sprouts during cold storage [29]. Despite these advances, the specific impact of pre-harvest 1-MCP application or precooling on the transcriptional regulation of chlorophyll biosynthetic and degradation pathways in broccoli remains poorly characterized, highlighting a critical need for systematic investigation.

Transcriptomic profiling via RNA sequencing (RNA-Seq) offers a powerful tool for uncovering genome-wide regulatory networks underlying postharvest physiology [30,31,32]. In this study, we performed RNA-Seq analysis to compare the effects of preharvest 1-MCP treatment (simulating a chemically preserved ambient transport) versus the ideal pre-cooling and cold chain storage. Our work seeks to elucidate how preharvest 1-MCP priming functions as a potential alternative to cold chain logistics by identifying key differentially expressed genes and senescence-related pathways, thereby providing a molecular basis for optimizing preservation protocols for broccoli when cold chain facilities are unavailable.

## 2. Materials and Methods

### 2.1. Plant Material and Treatments

Broccoli (*Brassica oleracea* var. *italica*, “Suqing 6”) was grown on a vegetable farm in Xiangshui County, Yancheng, China. For the preharvest 1-MCP-treated group (MG), plants were sprayed with 400 μL/L 1-MCP (Fresh Peak^®^ 1-MCP stable solution, Shandong Nutrition Source Food Technology Co., Ltd., Jinan, China) three days prior to harvest until run-off. Broccoli heads were harvested at commercial maturity and transported to the laboratory within 1 h. Upon arrival, heads free from mechanical damage and disease were selected and allocated into three experimental groups. The control group (CG) was established to simulate a commercial scenario where no low-temperature treatment is applied, reflecting typical challenges faced during postharvest handling; therefore, these heads were stored directly in cardboard boxes at 20 ± 1 °C and 70% relative humidity (RH) (simulated ambient transportation). Similarly, the MG samples, following the preharvest spray described above, were packed in cardboard boxes and stored under the same simulated ambient transportation conditions (20 ± 1 °C, 70% RH). In contrast, the pre-cooling followed by the cold-chain group (PG) represented an ideal scenario where harvested broccoli is subjected to immediate pre-cooling followed by cold storage; specifically, the heads were subjected to rapid pre-cooling at 4 °C for 6 h immediately after transport, and subsequently stored unpackaged at 0 ± 1 °C and 70% RH.

### 2.2. Sampling and RNA Extraction

Broccoli florets were sampled at specific time points: day 0 for the control (CG-0) and day 1 for all groups (CG-1, MG-1, and PG-1). Samples were immediately frozen in liquid nitrogen and stored at −80 °C for transcriptomic analysis. Each experimental group included three biological replicates, with each replicate consisting of a pool of 30 florets randomly collected from three different heads.

### 2.3. Evaluation of Sensory Quality

Sensory evaluation was conducted by a panel of 10 trained assessors using a modified method described by Jiang et al. [6]. A trained panel consisting of 10 assessors (5 males and 5 females, aged 25–45 years) with prior experience in vegetable sensory analysis participated in 4 training sessions over a 2-week period. Sensory quality was assessed on a 9-point scale: score 9: intense green color, high freshness, tightly packed florets, high firmness, and no surface defects; score 7: good freshness, uniform green color, compact head, satisfactory firmness, and no off-odors; score 5: moderate greenness, slight loss of firmness, mildly loose florets, and no off-odors (marketable limit); score 3: noticeable freshness decline, visible yellowing (<30%), reduced firmness, and slight off-odor (unmarketable); score 1: extensive yellowing, severe floret detachment, distinct off-odors, and decay. All samples were coded with random 3-digit numbers under standardized fluorescent lighting (6500 K) to ensure blinding. The evaluations were performed in individual sensory booths maintained at 25 ± 2 °C. Each sample was evaluated in triplicate, and the average value was taken as the final result.

### 2.4. RNA Sequencing and Gene Expression Analysis

RNA-Seq was performed by Biomarker Technologies Co., Ltd. (Beijing, China). The total RNA was extracted using Trizol reagent (Invitrogen, Carlsbad, CA, USA) according to the manufacturer’s protocol. Sequencing libraries were prepared using the NEBNext^®^ Ultra^TM^ RNA Library Prep Kit for Illumina^®^ (New England Biolabs, Ipswich, MA, USA) following the manufacturer’s instructions. After purification and index coding, libraries were sequenced on an Illumina HiSeq X Ten platform (Illumina, Inc., San Diego, CA, USA). Gene expression levels were quantified using the FPKM (fragments per kilobase of transcript per million mapped fragments) method. Differential expression analysis was conducted using the DESeq2 R package (Version 1.38.0). Differentially expressed genes (DEGs) were identified with a threshold of |log2Foldchange| ≥ 1 and a false discovery rate (FDR) of <0.05. All experiments were performed in triplicate. Normality and homoscedasticity assumptions were not formally tested, as DESeq2 is based on the negative binomial distribution and does not require these assumptions for differential expression analysis.

To explore the biological functions of the identified DEGs, Gene Ontology (GO) enrichment analysis was conducted using the clusterProfiler R package (version 4.6.2) with the Benjamini–Hochberg FDR correction. Additionally, KEGG pathway enrichment of the DEGs was evaluated using KOBAS 3.0 software. Pathways with an FDR of < 0.05 were considered significantly enriched.

### 2.5. Statistical Analysis

The data are presented as mean ± standard deviation. Differences among groups were evaluated by one-way analysis of variance (ANOVA) followed by Duncan’s Multiple Range Test using SPSS software (Version 26.0, IBM Corp., Armonk, NY, USA). A *p*-value < 0.05 was considered statistically significant.

## 3. Results

### 3.1. Sensory Quality of Broccoli

Table 1 presents the changes in sensory quality of broccoli under different treatments. On day 0, all groups exhibited excellent quality with a score of 9.0. By day 1, the CG showed a significant decline in sensory quality, dropping to 7.4 ± 0.63 (*p* < 0.05). In contrast, both the PG and MG treatments effectively preserved the freshness of the broccoli, with sensory scores of 9.0 ± 0.00 and 8.7 ± 0.49, respectively. Notably, as indicated by the statistical letters, there was no significant difference between the PG and MG on day 1. This result demonstrates that, during the initial stage of simulated transportation, the MG was as effective as PG in maintaining the sensory quality of broccoli.

### 3.2. Transcriptome Sequencing and Sequence Quality

To comprehensively profile the transcriptomic changes in broccoli responding to 1-MCP and pre-cooling combined with cold treatments, twelve cDNA libraries were constructed from four groups of samples: control at 0 d (CG-0), control at 1 d (CG-1), 1-MCP-treated at 1 d (MG-1), and pre-cooling combined with cold at 1 d (PG-1). The sequencing generated a total of 79.74 Gb of clean data (Table 2). The Q30 percentage was consistently above 93.49%, and the GC content ranged from 46.96% to 47.68%, indicating high sequencing quality. Furthermore, the alignment statistics (Table 3) showed that more than 92.29% of the clean reads were successfully mapped to the broccoli reference genome, with unique mapping rates exceeding 88.81%. Gene expression levels were quantified based on the FPKM. The density distributions of the FPKM values (Supplementary Figure S1) displayed consistent patterns across biological replicates, demonstrating high reproducibility and validating the dataset’s reliability for subsequent differential gene expression analysis.

### 3.3. Identification of DEGs

The DEGs were identified using thresholds of |log2Foldchange| ≥ 1 and *p* < 0.05 [33]. The number of DEGs identified in each experimental group relative to the initial day 0 control (CG-0) is summarized in Table 4. The ambient storage control (CG-1) exhibited substantial transcriptional changes compared to CG-0, with a total of 11,889 DEGs identified (5812 upregulated and 6077 downregulated). Similarly, the 1-MCP-treated group (MG-1) showed extensive transcriptional reprogramming, exhibited the highest number of DEGs (12,235 total; 6030 upregulated and 6205 downregulated). In contrast, the pre-cooling combined with the cold-chain group (PG-1) displayed a markedly more stable transcriptome, with substantially fewer DEGs identified (3561 total; 1700 upregulated and 1861 downregulated). This pronounced difference indicates that the cold-chain treatment effectively suppressed global transcriptional fluctuations, maintaining a gene expression profile closer to that of fresh broccoli, whereas 1-MCP treatment under ambient conditions involved extensive transcriptional reprogramming.

**Figure 1:**
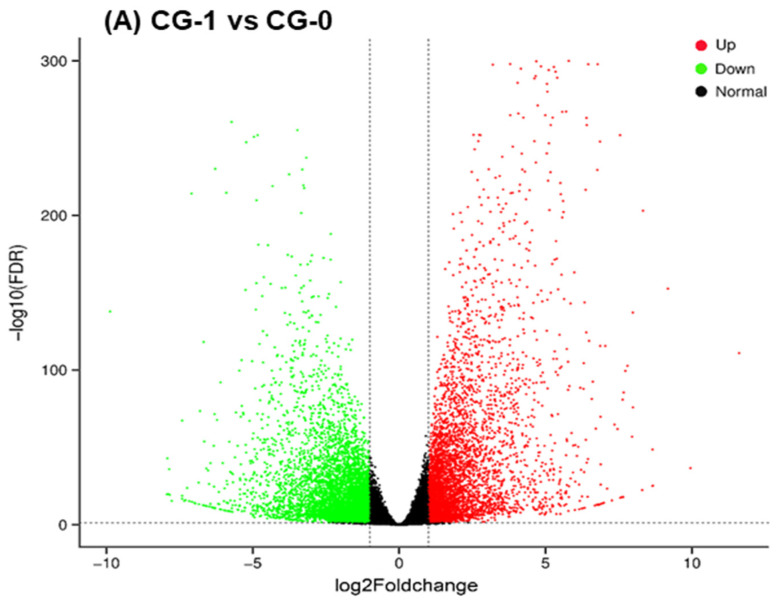

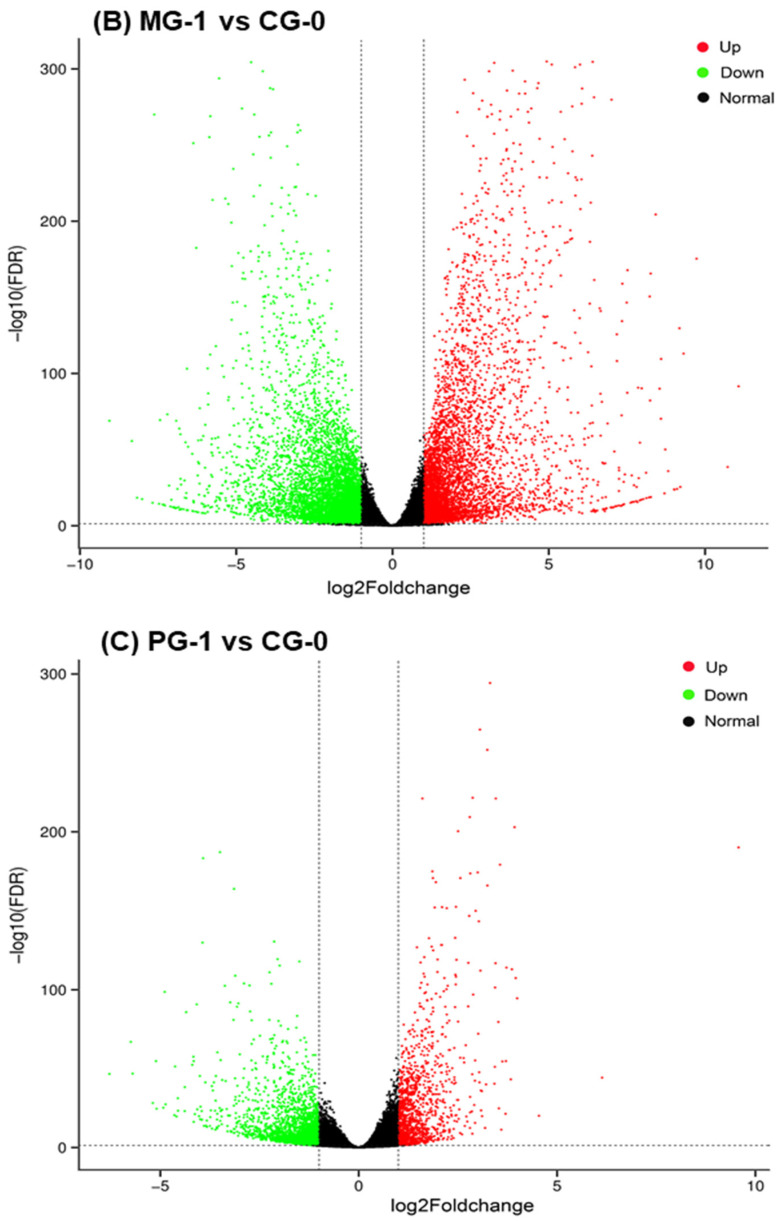
The volcano plots showing differentially expressed genes (DEGs) in response to different treatments. The *X*-axis represents the Log2Foldchange in gene expression, and the *Y*-axis represents the −log10 False discovery rate (FDR). The red dots indicate significantly upregulated genes (FDR < 0.05 and Log2Foldchange > 1), green dots indicate significantly downregulated genes (FDR < 0.05 and log2Foldchange < −1), and black dots represent non-significantly expressed genes. Comparisons are shown for: (**A**) CG-1 vs. CG-0, (**B**) MG-1 vs. CG-0, and (**C**) PG-1 vs. CG-0. The vertical and horizontal dashed lines indicate the filtering thresholds for Log2Foldchange and FDR, respectively.

Volcano plots for these comparisons are presented in Figure 1, visualizing the distribution of genes based on expression change and statistical significance. The distributions of up- and downregulated DEGs were approximately symmetrical across all comparisons. The majority of the DEGs exhibited |log2FoldChange| values between one and five, with only a small proportion showing |log2Foldchange| values greater than five.

### 3.4. GO Classification and Functional Enrichment of the DEGs

**Figure 2:**
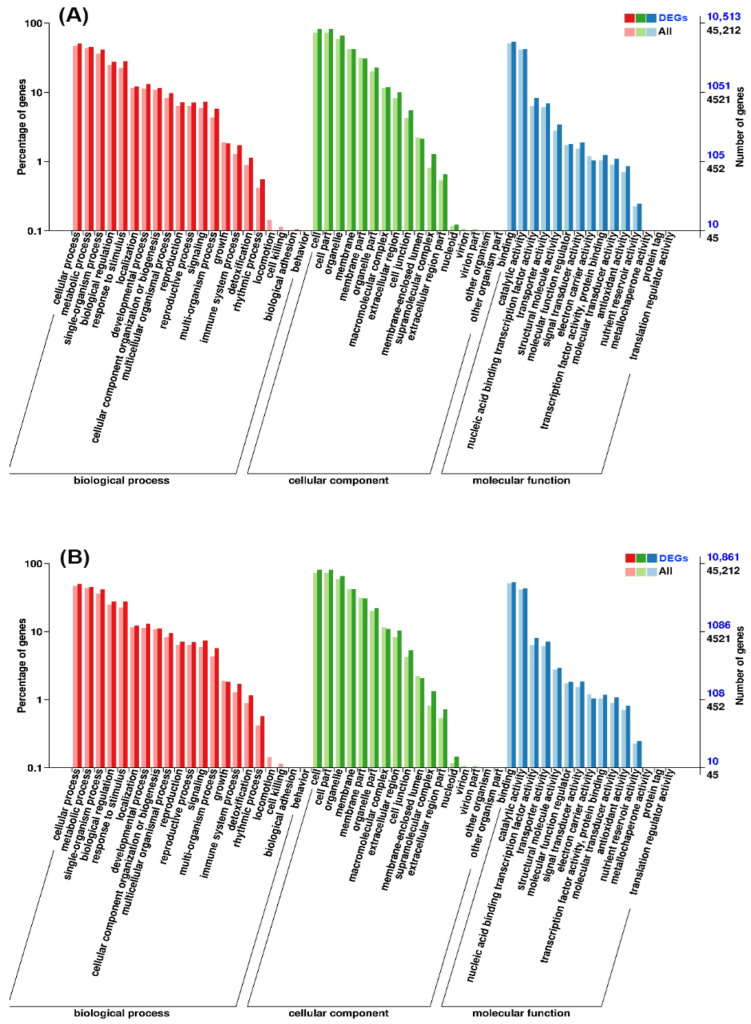

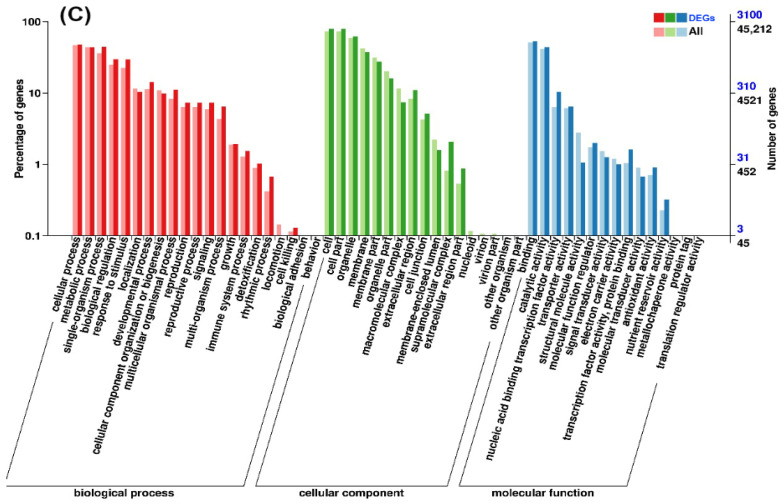
Gene Ontology (GO) classification of differentially expressed genes (DEGs) in each comparison group. The histograms show the distribution of DEGs (dark bars) compared with the background genome (all of the light bars) across three GO domains: biological process, cellular component, and molecular function. The left *Y*-axis indicates the percentage of genes within each category (calculated as the number of genes in the category divided by the total number of genes in the same GO domain for the respective gene set), and the right *Y*-axis indicates the absolute number of genes. Comparisons are shown for: (**A**) CG-1 vs. CG-0, (**B**) MG-1 vs. CG-0, and (**C**) PG-1 vs. CG-0 comparisons. (The background genome represents all annotated genes in the reference genome).

To understand the biological functions associated with the transcriptional changes, the identified DEGs were assigned to 52 functional categories across the three main Gene Ontology (GO) domains: biological process, cellular component, and molecular function. As shown in Figure 2, within the biological process domain, “cellular process,” “metabolic process,” and “single-organism process” were the most abundant terms. For the cellular component, the majority of DEGs were categorized under “cell” and “cell part,” while “catalytic activity” and “binding” were the predominant terms in the molecular function category.

We next performed GO enrichment analysis to identify the specific biological pathways affected by the different treatments. In the MG-1 vs. CG-0 comparison, the most significantly enriched biological processes included the “cellulose metabolic process” and the “carotenoid biosynthetic process,” suggesting that the 1-MCP treatment induced active regulation of cell wall remodeling and secondary metabolism even under ambient conditions. In contrast, for the PG-1 vs. CG-0 comparison, significant enrichments were observed in the cellular component “plasma membrane” and biological processes such as the “fatty acid biosynthetic process” and the “cellulose metabolic process.” Notably, enrichment in cell growth-related terms (e.g., “root hair elongation”) was also observed. These results indicate that the pre-cooling combined with the cold treatment primarily maintained membrane integrity and basic cell growth potential, thereby preserving the fresh-like state of the broccoli (Supplementary Figure S3).

### 3.5. KEGG Pathway Classification and Enrichment Analysis of the DEGs

The DEGs identified across all comparisons were classified into 50 categories based on the KEGG pathway database (Supplementary Figure S4).

**Figure 3:**
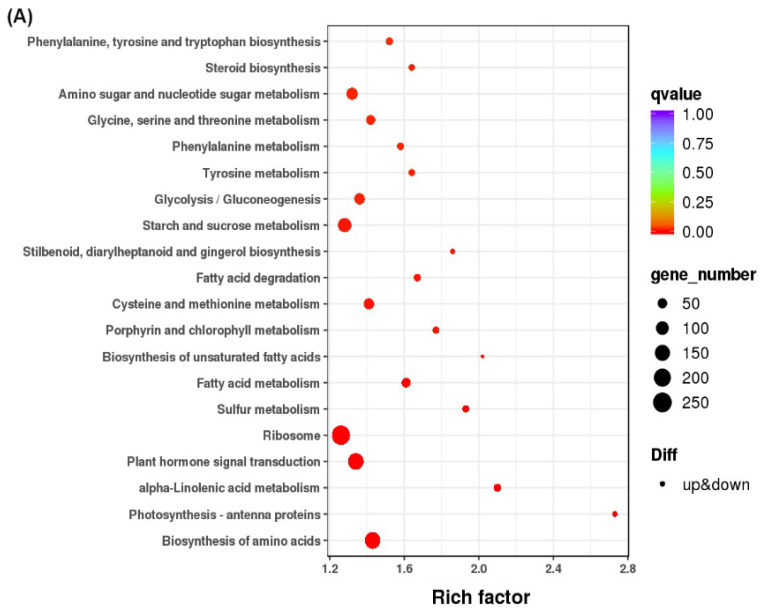

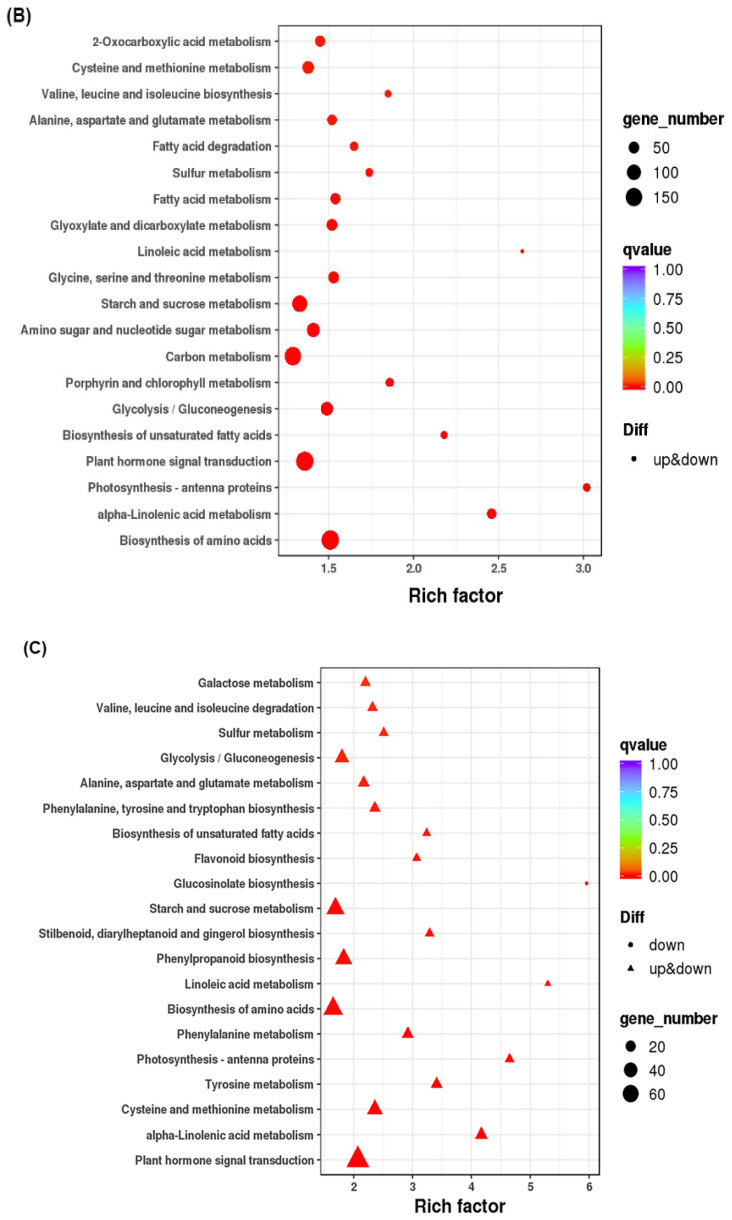
KEGG pathway enrichment analysis of differentially expressed genes (DEGs) in each comparison group. The bubble plots show enriched KEGG pathways for (**A**) CG-1 vs. CG-0, (**B**) MG-1 vs. CG-0, and (**C**) PG-1 vs. CG-0. The *Y*-axis lists the pathway names, and the *X*-axis represents the rich factor (the ratio of the number of DEGs annotated to a pathway to the total number of annotated genes in that pathway). The size of each bubble corresponds to the number of DEGs mapped to the pathway, and the color gradient represents the statistical significance (q-value), with darker colors indicating more significant enrichment (lower q-values). Pathways with q < 0.05 were considered significantly enriched.

In the CG-1 vs. CG-0 comparison, 53.3% of the DEGs were mapped to “Metabolism”, followed by “Genetic Information Processing” (27.9%) and “Environmental Information Processing” (8.9%). Pathway enrichment analysis (Figure 3A) indicated that “Ribosome” was the most significant pathway. Additionally, pathways related to energy and metabolism, such as “Photosynthesis-antenna proteins”, “Biosynthesis of amino acids”, and “Fatty acid metabolism”, as well as “Plant hormone signal transduction”, were also significantly enriched.

For the MG-1 vs. CG-0 comparison (Figure 3B), the DEGs were mainly distributed in “Metabolism” (56.1%), “Genetic Information Processing” (24.9%), and “Environmental Information Processing” (9.0%). Enrichment analysis highlighted “Biosynthesis of amino acids” and “Plant hormone signal transduction” as the most prominent pathways, alongside “Carbon metabolism” and “Starch and sucrose metabolism”.

Notably, in the PG-1 vs. CG-0 comparison, the proportion of DEGs assigned to “Metabolism” increased to 66.9% (Supplementary Figure S4), while “Genetic Information Processing” decreased to 13.9%, and “Environmental Information Processing” accounted for 11.7%. Enrichment analysis (Figure 3C) revealed that “Plant hormone signal transduction” and “Biosynthesis of amino acids” were the most significantly enriched. Furthermore, stress response and secondary metabolism pathways, including “alpha-Linolenic acid metabolism”, “Phenylpropanoid biosynthesis”, and “Starch and sucrose metabolism”, showed significant enrichment, suggesting a complex metabolic adaptation in this group.

### 3.6. Transcriptome Analysis of DEGs for Related Enzymes in Chlorophyll Metabolism

Surface color is a key visual indicator of broccoli quality. Transcriptome analysis identified a total of 27 DEGs annotated as key enzymes involved in chlorophyll metabolism across the CG-1, MG-1, and PG-1 groups compared to CG-0. Among them, the numbers of up/downregulated DEGs were 9/17 in CG-1, 8/14 in MG-1, and 4/6 in PG-1, respectively. The detailed annotation information of DEGs in the three comparisons can be found in Supplementary Table S1.

**Figure 4:**
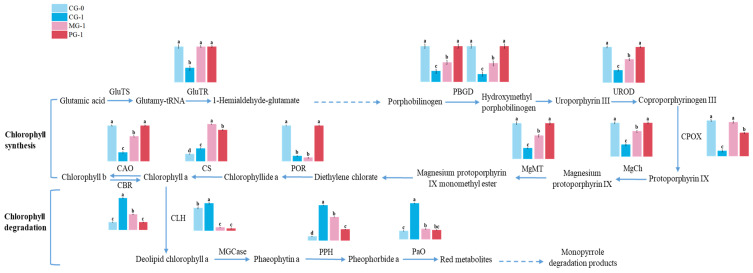
A schematic diagram of the chlorophyll metabolic pathway and relative expression of key genes in broccoli under 1-MCP and pre-cooling combined with cold treatments. Given the complexity of the pathway, enlarged and detailed expression profiles with statistical analysis for all genes are provided in Supplementary Figure S5. Different lowercase letters above the bars indicate significant differences among treatments (*p* < 0.05). Dashed arrows represent indirect reactions involving multiple enzymatic steps.

Chlorophyll metabolism comprises synthesis, recycling, and degradation [34]. The chlorophyll metabolic pathway and key enzyme expression patterns are illustrated in Figure 4. We analyzed thirteen key enzymes: eight involved in synthesis, two in recycling, and three in degradation.

Glutamyl-tRNA reductase (GluTR) initiates chlorophyll synthesis (Figure 4; Supplementary Figure S5A). In the untreated control (CG-1), the expression of GluTR genes dropped significantly compared to day 0 (CG-0). The 1-MCP treatment significantly improved the expression of three GluTR genes (*BolC1t03562H*, *BolC8t52456H*, and *BolC1t03172H*) but not in *BolC2t11967H*. Similarly, the pre-cooling combined with cold treatment effectively maintained high expression levels of these three genes, although it failed to prevent the downregulation of *BolC2t11967H*. Notably, *BolC8t52456H* showed low expression in both controls but was significantly induced by both treatments.

GluTR (glutamyl-tRNA reductase) serves as the rate-limiting enzyme in the tetrapyrrole biosynthetic pathway, catalyzing the conversion of glutamyl-tRNA to glutamate-1-semialdehyde, which is a pivotal step for chlorophyll and heme synthesis [35]. The functional importance of GluTR is well-established, with recent studies highlighting its regulation by the GluTR-binding protein (GBP) and its involvement in stress adaptation, thereby providing high confidence in its pivotal role [36]. *BolC1t03562H* is a BolC-specific gene with currently moderate confidence regarding its function; it is annotated in the Bolbase database and is likely involved in stress response pathways, consistent with the enrichment of stress-responsive genes in the C subgenome [37]. *BolC8t52456H* also belongs to the C subgenome and is hypothesized to participate in secondary metabolism, potentially influencing glucosinolate or flavonoid biosynthesis—processes that are often C-subgenome-biased. *BolC2t11967H* shows similarity to NLR (nucleotide-binding leucine-rich repeat) disease-resistant genes, suggesting a role in pathogen defense; this aligns with recent observations of NLR gene clusters preferentially retained in the C subgenome of the Brassica species [38]. *BolC9t59612H* is predicted to encode a metabolic enzyme, possibly linked to lipid or carbohydrate metabolism, although functional validation remains pending, thus warranting low confidence at this stage.

Both treatments sustained the expression of downstream synthesis enzymes (Figure 4; Supplementary Figure S5B), including porphobilinogen deaminase (PBGD) (*BolC3t12952H*, *BolC9t59612H*) and uroporphyrinogen III decarboxylase (UROD) (*BolC4t28337H*), effectively counteracting the downregulation observed in CG-1. Notably, for coproporphyrinogen-III oxidase (CPOX) (*BolC8t52801H*), the 1-MCP treatment induced a stronger upregulation compared to the pre-cooling combined with the cold treatment.

Further downstream (Figure 4; Supplementary Figure S5C), genes encoding magnesium chelatase subunits (MgCh D/H/I) and magnesium protoporphyrin IX methyltransferase (MgMT) were significantly suppressed in CG-1 but were well-maintained in both treatment groups. Interestingly, the pre-cooling combined with the cold treatment (PG-1) often maintained higher expression levels of MgCh genes (e.g., *BolC5t29422H*) than the 1-MCP treatment.

In the final steps of biosynthesis involving protochlorophyllide oxidoreductase (POR), chlorophyll synthase (CS), and chlorophyllide a oxygenase (CAO) (Figure 4; Supplementary Figure S5D), both treatments generally maintained expression levels comparable to CG-0. A notable exception was the CS gene (*BolC8t50320H*), which was not merely maintained but significantly upregulated by both treatments to levels exceeding those in CG-0. Conversely, regarding the POR gene *BolC1t02306H*, the 1-MCP treatment failed to prevent its downregulation, whereas the pre-cooling combined with the cold treatment successfully maintained its expression levels comparable to CG-0. This differential regulation suggests a potential functional divergence between these POR isoforms. The functional role of *BolC1t02306H* in chlorophyll metabolism is supported by evolutionary conservation and prior functional studies. In *Arabidopsis*, loss-of-function mutations in the homologous gene CLH1 result in delayed chlorophyll degradation during leaf senescence, directly demonstrating the catalytic role of chlorophyllase in hydrolyzing chlorophyll molecules. *BolC1t02306H* serves as a key chlorophyll catabolic enzyme in broccoli, consistent with its annotated protein domain architecture containing the conserved α/β hydrolase motif characteristic of chlorophyllase family members.

The specific role and validation of *BolC1t02306H* requires further investigation, as its expression might be regulated by distinct mechanisms or factors not addressed in our experimental setup.

Regarding chlorophyll degradation (Figure 4; Supplementary Figure S5E), genes encoding chlorophyll b reductase (CBR), chlorophyllase (CLH), pheophytinase (PPH), and pheophorbide a oxygenase (PaO) were drastically upregulated in the senescing control (CG-1). Both treatments significantly inhibited this upregulation. Crucially, the pre-cooling combined with the cold treatment demonstrated a superior inhibitory effect, suppressing the expression of key degradation genes—particularly PPH (*BolC1t02539H*) and CBR (*BolC8t47700H*)—more effectively than the 1-MCP treatment.

In summary, both treatments delayed yellowing by maintaining the expression of chlorophyll synthesis genes and suppressing degradation genes. The pre-cooling combined with the cold treatment was particularly effective in repressing the degradation pathway, while 1-MCP showed distinct strengths in sustaining specific synthesis genes like CPOX.

Beyond chlorophyll metabolism, KEGG pathway enrichment analysis (Supplementary Figure S4) revealed that DEGs were also significantly represented in broader physiological processes. In both MG-1 and PG-1 groups, genes involved in “Phenylpropanoid biosynthesis” and “MAPK signaling pathway–plant” showed differential regulation compared to the CG-1 control. Notably, several genes encoding antioxidant enzymes, such as peroxidase (POD) and superoxide dismutase (SOD), maintained higher transcript levels in the 1-MCP and pre-cooling treated samples. Alongside the sustained expression of vital downstream synthesis enzymes like PBGD (*BolC3t12952H*, *BolC9t59612H*), these findings suggest that the preservation of broccoli quality is a systemic response involving not only the inhibition of chlorophyll degradation but also the reinforcement of secondary metabolism and overall cellular antioxidant capacity.

## 4. Discussion

Previous studies have demonstrated that both 1-MCP and pre-cooling combined with cold treatments are effective in maintaining the quality of broccoli by reducing chlorophyll degradation and inhibiting floret yellowing and senescence [2]. In this study, transcriptome analysis of broccoli treated with 1-MCP and pre-cooling combined with the cold treatment was performed to gain deeper insights into their effects on gene expression during a one-day transport simulation.

It should be noted that while the initial temperature trajectory differed between the preharvest 1-MCP (MG) and rapid pre-cooling (PG) groups, this study was designed to evaluate two distinct, holistic postharvest management strategies rather than isolating temperature as a single variable. The pre-cooling combined with the cold treatment represents an idealized physical intervention (immediate heat removal), whereas the preharvest 1-MCP treatment mimics a realistic chemical alternative used to compensate for potential delays in the cold chain, which is a common logistical challenge. Our comparative transcriptomic analysis suggests that preharvest 1-MCP application acts as a physiological buffer, allowing the broccoli to better tolerate the slower temperature decline during transport by altering its baseline ethylene sensitivity.

The GO enrichment analysis of the DEGs revealed that 1-MCP significantly affected photosynthetic processes in broccoli. Cytokinins are a class of plant hormones that regulate plant growth, development, and differentiation. Downs et al. reported that cytokinin treatment can alter the physiological changes associated with the yellowing of broccoli florets [39]. In our study, 1-MCP treatment significantly enhanced the response to cytokinin, a finding consistent with reports of 1-MCP action in peaches [40]. Furthermore, the KEGG enrichment analysis indicated that 1-MCP treatment influences carbon metabolism in broccoli. As a fundamental physiological process, carbon metabolism is crucial for the quality maintenance of postharvest broccoli. Supporting this, Xu et al. reported that 1-MCP treatment enhances the activity of sucrose synthase and concurrently induces the expression of genes encoding sucrose transporters (*BoSUC1* and *BoSUC2*) and key carbohydrate-metabolizing enzymes (*BoINV1*, *BoHK1*, and *BoHK2*) [8]. Therefore, we propose that the regulation of carbon metabolism is one mechanism by which 1-MCP maintains broccoli quality during transport.

### 4.1. DEGs Involved in Chlorophyll Biosynthesis After 1-MCP and Pre-Cooling Combined with Cold Treatments

In our previous study [41], the chlorophyll content of broccoli decreased in all samples during the one-day transport simulation. However, pre-cooling combined with the cold and 1-MCP treatments delayed this loss, with treated florets maintaining significantly higher chlorophyll levels than the controls. Specifically, the chlorophyll degradation rate in the control group reached 15.98% by day one, which is substantially higher than the 1.36% and 6.97% observed in the pre-cooling followed by the cold and 1-MCP groups, respectively. Concurrently, chlorophyllase activity increased by 4.53% in the control group, compared to only 0.67% and 0.91% in the pre-cooling combined with cold and 1-MCP groups, respectively. Pearson correlation analysis in that study revealed a significant negative correlation between the chlorophyll content and chlorophyllase activity (*r* = −0.747, *p* < 0.01). These physiological results demonstrate that both treatments effectively suppress chlorophyllase activity and delay chlorophyll degradation.

While past research has predominantly focused on how 1-MCP and cold storage regulate chlorophyll degradation, their impact on chlorophyll biosynthesis remains less explored. Vavilin et al. reported that while massive chlorophyll breakdown mediated by chlorophyllase occurs during senescence, active chlorophyllases are present in tissues of all ages, indicating that hydrolysis does not always lead to a net chlorophyll loss [42]. Our transcriptomic data provide a more comprehensive view: we identified nine DEGs involved in chlorophyll biosynthesis and four in degradation. The nine biosynthesis-related genes (one DEG corresponds to two genes) were predominantly upregulated or maintained, while the four degradation-related genes were downregulated by the treatments. This coordinated gene expression suggests that 1-MCP and pre-cooling combined with cold treatments maintain the chlorophyll content through a dual mechanism: enhancing biosynthesis while simultaneously suppressing degradation.

Chlorophyll biosynthesis is a multi-step enzymatic process. Glutamyl-tRNA reductase (GluTR), a key rate-limiting enzyme, catalyzes the reduction of glutamyl-tRNA to glutamate-1-semialdehyde (GSA), initiating the pathway [43]. In this study, 1-MCP treatment significantly upregulated or maintained the expression of three GluTR genes (*BolC1t03562H*, *BolC8t52456H*, *BolC1t03172H*) compared to the control (CG-1). However, neither treatment could prevent the downregulation of *BolC2t11967H*. Interestingly, *BolC8t52456H* showed a unique pattern, being strongly induced by both treatments despite low expression in the initial control.

Subsequently, porphobilinogen deaminase (PBGD), uroporphyrinogen decarboxylase (UROD), and coproporphyrinogen oxidase (CPOX) are crucial for the synthesis of protoporphyrinogen IX [44]. Both treatments sustained high expression levels of PBGD (*BolC9t59612H*, *BolC3t12952H*), UROD (*BolC4t28337H*), and CPOX (*BolC8t52801H*). Notably, 1-MCP exerted a stronger upregulatory effect on CPOX (*BolC8t52801H*) than the pre-cooling combined with cold treatment.

Magnesium chelatase (MgCh) and magnesium protoporphyrin IX methyltransferase (MgMT) are pivotal enzymes that catalyze the formation of Mg-protoporphyrin IX and Mg-protoporphyrin IX monomethyl ester, respectively. MgCh is a multi-subunit complex (ChlH, ChlD, ChlI) that inserts Mg^2+^ into protoporphyrin IX [45]. Our data show that both treatments maintained elevated expression of MgCh and MgMT genes. As Beale et al. noted, active MgCh directs protoporphyrin IX toward chlorophyll synthesis and away from heme production [44]. Therefore, we propose that the upregulation of MgCh by these treatments promotes chlorophyll formation.

Protochlorophyllide oxidoreductase (POR) catalyzes the penultimate step of chlorophyll biosynthesis. This light-dependent enzyme also participates in a negative feedback loop and is critical for controlling chlorophyll levels [46]. In our study, 1-MCP sustained the expression of the POR gene *BolC8t46871H* but failed to prevent the downregulation of *BolC1t02306H*. This differential regulation suggests functional divergence between these POR isoforms. The specific role of *BolC1t02306H* requires further investigation, as it may be a pseudogene or expressed under specific conditions not captured here.

### 4.2. DEGs Involved in Chlorophyll Cycling After 1-MCP and Pre-Cooling Combined with Cold Treatments

The chlorophyll cycle, which occurs in the chloroplasts of higher plants, involves the interconversion of chlorophyll a and chlorophyll b. The forward reaction, catalyzed by chlorophyllide a oxygenase (CAO), converts chlorophyll a to chlorophyll b. The reverse reaction, catalyzed by chlorophyll b reductase (CBR), primarily converts chlorophyll b back to chlorophyll a during senescence or under other specific conditions [47]. In this study, 1-MCP promoted CAO expression while downregulating CBR genes. This expression pattern strongly suggests that 1-MCP regulates the chlorophyll cycle.

Chlorophyllide a oxygenase (CAO) catalyzes the oxidation of chlorophyllide a to chlorophyllide b, representing the committed step in chlorophyll b biosynthesis. This conversion fundamentally determines the chlorophyll a/b ratio, which is critical for the structural integrity of light-harvesting complexes and overall photosynthetic efficiency [48].

The chlorophyll a/b ratio serves as a valuable indicator of senescence progression. During natural aging, the preferential degradation of chlorophyll b via the chlorophyll b reductase pathway results in an elevated chlorophyll a/b ratio. 1-MCP treatment, through the coordinated regulation of CAO (upregulation) and CBR (suppression), maintains the relative stability of the chlorophyll a/b ratio. This stabilization not only preserves the structural integrity of light-harvesting complexes but may also constitute an active mechanism for delaying the senescence program.

The regulatory mechanism of 1-MCP in modulating chlorophyll metabolism represents a critical finding with broader implications for postharvest physiology. Our results align with previous transcriptomic studies demonstrating CAO upregulation during broccoli browning [49], a phenomenon hypothesized to disrupt metabolic homeostasis through dysregulated chlorophyll biosynthesis. Mechanistically, the competitive binding of 1-MCP to ethylene receptors (ETR/ERS family) inhibits downstream EIN3/EIL transcriptional activation, thereby alleviating the suppressive effect of ethylene signaling on CAO expression. This regulatory axis provides a molecular explanation for the observed delay in senescence-related chlorophyll degradation following 1-MCP treatment.

Furthermore, the preharvest application of 1-MCP (MG) in our study introduces an intriguing mechanistic dimension. Rather than acting solely as a passive postharvest preservation agent, preharvest 1-MCP likely functions as a “chemical priming” elicitor. By blocking ethylene perception while the broccoli is still attached to the plant and metabolically highly active, it pre-acclimates the tissue without triggering acute physiological damage (as evidenced by the lack of widespread acute stress biomarkers). This priming effect fortifies the plant’s basal defense mechanisms—such as the sustained expression of vital metabolic enzymes like PBGD (BolC3t12952H) and enhanced antioxidant capacity—before the acute stress of harvest and transport actually occurs. Consequently, the MG florets are pre-conditioned to withstand the subsequent logistical delays, acting as a proactive stress-mitigation strategy rather than just reactive preservation.

However, it is established that CAO accumulates and actively synthesizes chlorophyll b in response to low chlorophyll b levels as part of a homeostatic mechanism [47]. Supporting the importance of this cycle, transgenic plants overexpressing a truncated CAO gene to overproduce chlorophyll b exhibited delayed senescence and a downregulated expression of senescence-associated genes [50]. Therefore, by upregulating CAO and downregulating CBR, 1-MCP appears to maintain the homeostatic function of the chlorophyll cycle. This ensures the continued ability to synthesize chlorophyll b while limiting its degradation, a mechanism that aligns with the observed delay in senescence in CAO-overexpressing plants. The simultaneous upregulation of CAO and suppression of chlorophyll b reductase by 1-MCP treatment creates a synergistic effect that maintains chlorophyll b homeostasis and stabilizes the chlorophyll a/b ratio, preventing the rapid decline of chlorophyll b that typically accompanies senescence [48].

Beyond facilitating chlorophyll b and light-harvesting complexes degradation during senescence, the chlorophyll cycle is now recognized to have profound physiological significance, analogous to the xanthophyll cycle. Growing evidence suggests that the interconversion of Chl a and Chl b is not merely a reversible side reaction but plays critical and specific roles in plant physiology [51]. This perspective helps explain the findings of Luo et al., who attributed early broccoli yellowing to an imbalance in the chlorophyll cycle—specifically, an accumulation of chlorophyll b alongside a deficiency of chlorophyll a, coupled with overall chlorophyll deficiency and carotenoid accumulation [49]. Our results demonstrate that 1-MCP helps to prevent such detrimental imbalances. By inhibiting the ethylene signal transduction, 1-MCP downregulates downstream senescence-associated genes, including those encoding chlorophyll catabolic enzymes such as non-yellow coloring 1 (NYC1) and hydroxychlorophyll a reductase (HCAR). This suppression of the ethylene signaling pathway not only delays chlorophyll degradation but also reduces the overall senescence progression in the harvested florets. In pak choi (Brassica chinensis), transcription factors including *BcNAC055*, *BcMYB44*, and *BcOBF1* have been identified to directly bind the promoter of BcNYC1 and activate its expression. 1-MCP treatment suppresses the expression of these transcription factors, thereby indirectly modulating downstream chlorophyll metabolism genes [52]. A similar regulatory paradigm may govern CAO expression in broccoli florets.

While the present synthesis provides additional supporting data for understanding the 1-MCP-mediated regulation of chlorophyll metabolism, several limitations warrant acknowledgment. First, direct experimental evidence documenting CAO gene expression changes in 1-MCP-treated broccoli remains limited, with current inferences derived primarily from studies of other chlorophyll metabolism genes. Second, quantitative data on CAO protein levels and enzymatic activity following 1-MCP treatment have not been reported. Third, the coordinated regulatory mechanisms underlying the concurrent CAO upregulation and chlorophyll b reductase suppression require further elucidation. Future studies employing proteomic and metabolomic approaches will be essential for validating these regulatory relationships.

Furthermore, it is noteworthy that the delay in senescence afforded by 1-MCP and pre-cooling extends beyond the maintenance of chlorophyll homeostasis. The enrichment of the phenylpropanoid and antioxidant pathways (Supplementary Figure S4) suggests a reinforcement of cell wall structural integrity and an enhanced capacity to mitigate reactive oxygen species (ROS) triggered by harvest-induced stress. This systemic coordination between primary (chlorophyll) and secondary metabolism ensures holistic quality preservation during postharvest logistics.

### 4.3. DEGs Involved in Chlorophyll Degradation After 1-MCP and Pre-Cooling Combined with Cold Treatments

Studies have demonstrated that 1-MCP treatment significantly influences the expression patterns of genes involved in chlorophyll degradation. Chlorophyllase (CLH), pheophytinase (PPH), and phytoene desaturase (PaO) play significant roles in the chlorophyll degradation pathway. Our results demonstrate that both 1-MCP and pre-cooling combined with cold treatments inhibit chlorophyll degradation by downregulating the expression of CBR, CLH, PPH, and PaO. Notably, the suppressive effect of the pre-cooling combined with cold treatment on PPH and CBR expression was more pronounced than that of 1-MCP.

This regulatory mechanism is consistent with studies in other species. For instance, 1-MCP was shown to suppress the activities of chlorophyll-degrading enzymes and the accumulation of intermediate metabolites in pak choi [53]. Similarly, in pears, the expression of a PPH gene was stimulated by ethylene and suppressed by 1-MCP [54]. These findings, along with the established role of ethylene in upregulating chlorophyllase activity during broccoli senescence [55], collectively support our conclusion that both 1-MCP and pre-cooling combined with cold treatments in broccoli function, at least in part, by antagonizing ethylene-mediated chlorophyll degradation. This is further corroborated by Cai et al., who reported that ethylene profoundly influences the expression of chlorophyll degradation genes (e.g., PaO) in harvested broccoli [7].

The core enzymes of chlorophyll degradation, including CBR, PPH, PaO, and RCCR, are conserved across species such as *Arabidopsis* and rice [56,57]. It is well-established that CLH, MDCase, and PaO are crucial for this process [58]. Our study shows that the expression of these key enzymes (CLH, PaO, and PPH) was upregulated in the control group during transport. In contrast, both 1-MCP and pre-cooling followed by cold treatments maintained their low expression levels, with the trend for chlorophyllase (CLH) being consistent with our previous physiological data.

**Figure 5:**
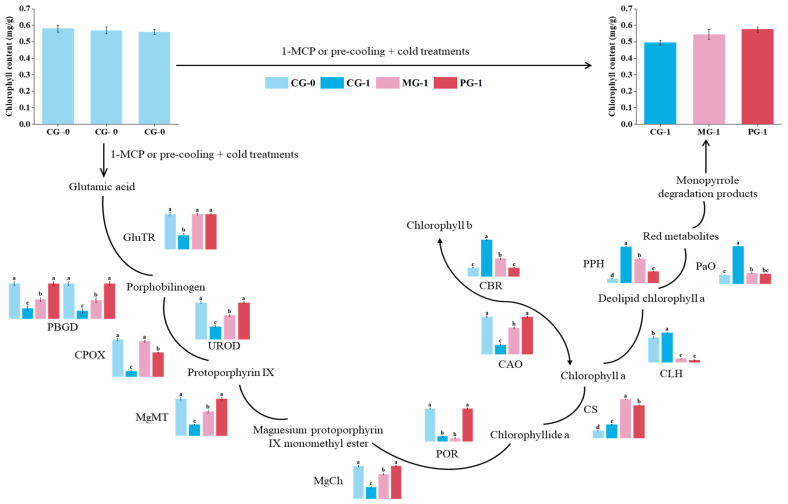
The proposed model for the green retention mechanism of 1-MCP and pre-cooling + cold treatments during broccoli transportation (1 day). The histograms display the expression levels of key genes and metabolite contents. CG-0 (light blue) represents the initial control at day 0. CG-1 (dark blue) represents the control group stored at an ambient temperature (20 °C) for 1 day. MG-1 (pink) represents the preharvest 1-MCP-treated group stored at an ambient temperature (20 °C) for 1 day. PG-1 (red) represents the pre-cooling combined with the cold-treated group stored at low temperature (0 °C) for 1 day. Different lowercase letters indicate significant differences (*p* < 0.05).

In conclusion, our study demonstrates that 1-MCP and pre-cooling combined with cold treatments maintain broccoli chlorophyll content by coordinately upregulating genes involved in chlorophyll biosynthesis while suppressing those responsible for its degradation. Based on these findings, we propose a comprehensive model (Figure 5) that delineates the molecular mechanism by which these treatments regulate chlorophyll metabolism to delay postharvest senescence.

Although qRT-PCR validation was not performed in the current study, the robustness of our transcriptomic data is strongly supported by stringent quality control metrics. Specifically, the sequencing mapping rates to the reference genome exceeded 92.29%, with uniquely mapped rates consistently above 88.81% across all samples (Table 3). Moreover, the FPKM density distributions demonstrated excellent consistency among biological replicates (Supplementary Figure S1). Furthermore, the differential expression profiles align closely with the observed macroscopic sensory evaluations, providing robust cross-validation at the phenotypic level. Nevertheless, future studies should incorporate qRT-PCR analysis to confirm the expression patterns of key chlorophyll metabolism genes identified through transcriptome sequencing, thereby strengthening the reliability of our proposed regulatory network. Moreover, functional validation through gene overexpression or silencing experiments in broccoli or model plant systems would help establish causal relationships between specific genes and the phenotypic outcomes of combined 1-MCP and cold treatments. Despite these limitations, our transcriptome-based model provides a valuable framework for understanding the molecular basis of chlorophyll retention in postharvest broccoli and identifies promising targets for future research aimed at developing more effective strategies to extend the shelf life of this important vegetable crop.

Our transcriptomic analysis provides compelling evidence that the differential expression of chlorophyll metabolism genes (DEGs) in broccoli florets is intricately linked to the modulation of ethylene signaling pathways. The coordinated upregulation of biosynthetic genes (e.g., GluTR, MgCh, POR) and the simultaneous downregulation of catabolic genes (e.g., CLH, PPH, PaO) in both 1-MCP and pre-cooling treatments suggest a dual regulatory mechanism that directly targets ethylene-mediated senescence signals.

1-MCP is known to competitively bind ethylene receptors (ETR/ERS family), effectively blocking the downstream activation of EIN3/EIL transcription factors that would otherwise upregulate senescence-associated genes, including those involved in chlorophyll degradation [59]. Our data align with this model, as the suppression of key catabolic genes (CLH, PPH, PaO) corresponds with the anticipated inhibition of ethylene signaling. Notably, the pre-cooling combined with the cold treatment exhibited an even stronger suppression of PPH and CBR expression, suggesting that low-temperature stress may further attenuate ethylene biosynthesis or signal transduction, possibly by affecting the stability of ethylene receptors or the activity of ACC oxidases. The upregulation of GluTR, MgCh, and POR genes indicates that ethylene signaling may also exert a suppressive effect on chlorophyll biosynthesis under normal senescence conditions. By blocking ethylene perception, 1-MCP likely releases this suppression, allowing the transcriptional activation of rate-limiting enzymes in the tetrapyrrole pathway. This is particularly evident in the sustained expression of MgCh subunits, which are known to direct a protoporphyrin IX flux towards chlorophyll synthesis rather than heme production [60]. The coordinated regulation of CAO (upregulated) and CBR (downregulated) further underscores the role of ethylene signaling in maintaining chlorophyll homeostasis. While CAO is typically induced under low chlorophyll b conditions, its upregulation in our study suggests that ethylene inhibition may mimic a low-b status, thereby activating a compensatory biosynthetic response. Conversely, the downregulation of CBR aligns with the notion that ethylene signaling promotes chlorophyll b degradation during senescence, and its inhibition stabilizes the chlorophyll a/b ratio [61].

Overall, the DEG patterns observed in our study provide molecular corroboration for the hypothesis that 1-MCP and pre-cooling treatments delay senescence primarily through the antagonism of ethylene signaling, thereby refocusing chlorophyll metabolism towards synthesis and maintenance rather than degradation. This integrated transcriptomic response highlights potential targets for future genetic or chemical interventions aimed at extending the postharvest shelf life of broccoli and similar horticultural crops.

### 4.4. Other Pathways Involved in Delaying Postharvest Senescence

In addition to chlorophyll metabolism, our KEGG enrichment analysis revealed that several other pathways were differentially regulated by the treatments. Notably, plant hormone signal transduction and alpha-linolenic acid metabolism (a key pathway for jasmonic acid biosynthesis) were significantly enriched in both MG-1 and PG-1 comparisons (Figure 3B,C). Additionally, pathways related to carbon metabolism, starch and sucrose metabolism, and phenylpropanoid biosynthesis showed differential enrichment patterns among the treatment groups. These results suggest that the quality maintenance effects of 1-MCP and pre-cooling treatments involve the coordinated regulation of multiple pathways beyond chlorophyll metabolism, including hormone signaling, secondary metabolism, and carbohydrate metabolism. The downregulation of alpha-linolenic acid metabolism, in particular, may contribute to reduced jasmonic acid production, thereby mitigating senescence-associated stress responses. Further investigation is warranted to elucidate the functional contributions of these pathways to delaying postharvest senescence.

## 5. Conclusions

In summary, this study employed RNA sequencing to investigate the effects of preharvest 1-MCP and pre-cooling combined with cold treatments on gene expression in broccoli. Transcriptome analysis revealed that both treatments significantly upregulated or maintained the key genes involved in chlorophyll biosynthesis (including GluTR, PBGD, UROD, CPOX, MgCh subunits, MgMT, POR, and CS) while downregulating the major genes responsible for chlorophyll degradation (such as CLH, PPH, and PaO). This coordinated gene regulation ultimately maintained higher chlorophyll content in the treated broccoli. Regarding the chlorophyll cycle, both treatments upregulated CAO expression and downregulated CBR expression. Collectively, these findings elucidate the molecular mechanisms by which 1-MCP and pre-cooling combined with cold treatments regulate chlorophyll metabolism in postharvest broccoli. This work provides new insights into the gene regulatory networks activated in response to these treatments.

## Figures and Tables

**Table 1:** The effects of preharvest 1-MCP and pre-cooling combined with cold treatments on the sensory quality of broccoli.

Storage Time (Day)	Sensory Score		
	CG	PG	MG
0	9.0 ± 0.00 ^a^ 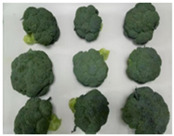		
1	7.4 ± 0.63 ^b^ 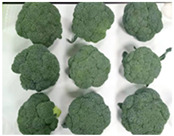	9.0 ± 0.00 ^a^ 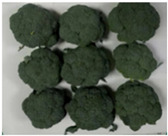	8.7 ± 0.49 ^a^ 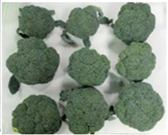

Different lowercase letters indicate significant differences among different treatments at the same storage time (*p* < 0.05).

**Table 2:** The sample sequencing data statistics.

Sample ID	Total Reads	Total Bases	GC (%)	Q30 (%)
CG-0-1	20,488,506	6,118,606,402	47.65	93.70
CG-0-2	22,205,091	6,625,875,124	47.67	93.70
CG-0-3	22,665,946	6,774,443,724	47.66	93.70
CG-1-1	23,778,443	7,111,056,580	47.04	94.07
CG-1-2	21,833,040	6,529,399,182	46.96	93.49
CG-1-3	19,883,695	5,936,890,730	47.00	94.09
MG-1-1	22,952,721	6,846,119,128	47.16	94.15
MG-1-2	25,461,310	7,592,535,476	47.27	93.65
MG-1-3	22,828,018	6,829,534,734	47.11	94.25
PG-1-1	22,572,903	6,744,483,548	47.64	93.91
PG-1-2	21,908,401	6,556,999,068	47.68	93.58
PG-1-3	23,146,928	6,916,454,632	47.53	93.76

Sample ID: Sample analysis number; total reads: total number of read pairs in clean data; total bases: total base number of clean data; GCcontent: percentage of the G and C bases in the total bases in clean data; and ≥Q30%: percentage of bases with sequencing quality scores greater than or equal to 30.

**Table 3:** The alignment statistics of the RNA-seq data to the reference genome.

Sample ID	Total Reads	Mapped Reads	Uniquely Mapped Reads	Multi-Mapped Reads	Reads Map to ‘+’	Reads Map to ‘−‘
CG-0-1	40,977,012	38,279,440 (93.42%)	37,197,622 (90.78%)	1,081,818 (2.64%)	19,057,678 (46.51%)	19,118,243 (46.66%)
CG-0-2	44,410,182	41,496,569 (93.44%)	40,368,316 (90.90%)	1,128,253 (2.54%)	20,657,158 (46.51%)	20,727,867 (46.67%)
CG-0-3	45,331,892	42,391,920 (93.51%)	41,210,029 (90.91%)	1,181,891 (2.61%)	21,110,265 (46.57%)	21,175,851 (46.71%)
CG-1-1	47,556,886	44,510,649 (93.59%)	43,294,196 (91.04%)	1,216,453 (2.56%)	22,162,467 (46.60%)	22,230,876 (46.75%)
CG-1-2	43,666,080	40,682,566 (93.17%)	39,510,192 (90.48%)	1,172,374 (2.68%)	20,243,850 (46.36%)	20,314,439 (46.52%)
CG-1-3	39,767,390	37,152,358 (93.42%)	36,109,474 (90.80%)	1,042,884 (2.62%)	18,490,899 (46.50%)	18,548,708 (46.64%)
MG-1-1	45,905,442	42,888,718 (93.43%)	41,700,776 (90.84%)	1,187,942 (2.59%)	21,338,858 (46.48%)	21,419,308 (46.66%)
MG-1-2	50,922,620	47,275,448 (92.84%)	45,769,419 (89.88%)	1,506,029 (2.96%)	23,505,373 (46.16%)	23,601,946 (46.35%)
MG-1-3	45,656,036	42,770,337 (93.68%)	41,605,581 (91.13%)	1,164,756 (2.55%)	21,287,487 (46.63%)	21,355,125 (46.77%)
PG-1-1	45,145,806	42,182,551 (93.44%)	40,993,645 (90.80%)	1,188,906 (2.63%)	20,997,932 (46.51%)	21,067,225 (46.66%)
PG-1-2	43,816,802	40,757,271 (93.02%)	39,543,067 (90.25%)	1,214,204 (2.77%)	20,283,187 (46.29%)	20,365,251 (46.48%)
PG-1-3	46,293,856	42,726,460 (92.29%)	41,115,615 (88.81%)	1,610,845 (3.48%)	21,241,099 (45.88%)	21,339,665 (46.10%)

Sample ID: sample analysis number; total reads: total number of paired-end reads in clean data; mapped reads: number of reads aligned to the reference genome and the percentage of clean reads; uniquely mapped reads: number of reads aligned to the unique location of the reference genome and the percentage of clean reads; multi-mapped reads: number of reads aligned to multiple locations in the reference genome and their percentage of clean reads; reads map to ‘+’: number of reads aligned to the positive strand of the reference genome and the percentage of clean reads; reads map to ‘−‘: number of reads aligned to the negative strand of the reference genome and the percentage of clean reads.

**Table 4:** The number of all the DEGs identified in each experimental group.

DEG Set	DEG Number	Upregulated	Downregulated
CG-1_vs_CG-0	11,889	5812	6077
MG-1_vs_CG-0	12,235	6030	6205
PG-1_vs_CG-0	3561	1700	1861

## Data Availability

The original contributions presented in this study are included in the article/Supplementary Materials. Further inquiries can be directed to the corresponding author.

## Supplementary Materials

The following supporting information can be downloaded at: https://www.mdpi.com/article/10.3390/foods15101688/s1, Figure S1: Comparison of FPKM density distribution of each sample; Figure S2: Heatmap of expression correlation among three groups; Figure S3: Functional enrichment analysis of DEGs; Figure S4: KEGG pathway classification of DEGs in three comparisons; Figure S5: Detailed relative expression profiles of all identified key genes involved in chlorophyll metabolism; Table S1: The detailed annotation information of DEGs in three comparisons.
