# Supplementary material for: Comparative Transcriptomic Analysis of Chlorophyll Metabolism in Broccoli Under Preharvest 1-MCP Application Versus Pre-Cooling Combined with Cold Chain Storage
Source: Foods. 2026 May 12;15(10):1688. doi: 10.3390/foods15101688 (PMC13205192; doi:10.3390/foods15101688)
Supplement: Supplementary file 1 [file foods-15-01688-s001.zip › Supplementary Figures.pdf]

Comparative Transcriptomic Analysis of Chlorophyll Metabolism in Broccoli Under Preharvest 1-MCP Application Versus Pre-Cooling Combined with Cold Chain Storage

Li Zhang [1,†], Tengfei Liu [2,†], Yingying Zhu [1], Libin Wang [3], Xiaoyu Xie [3] and Li Jiang [3,*]

[1] Food & Medicine Homology Big Health Innovation Consortium, Suzhou Polytechnic University, Suzhou 215104, China; zhangli_szd@163.com (L.Z.); 01412@jssvc.edu.cn (Y.Z.)
[2] Jiangsu Taihu Area Institute of Agricultural Sciences, Suzhou 215106, China; liutengfei@jaas.ac.cn
[3] College of Food Science and Technology, Nanjing Agricultural University, Nanjing 210095, China; lbwang88@126.com (L.W.); nau_xxiaoyu@163.com (X.X.)
* Correspondence: jiangli@njau.edu.cn
† These authors contributed equally to this work.

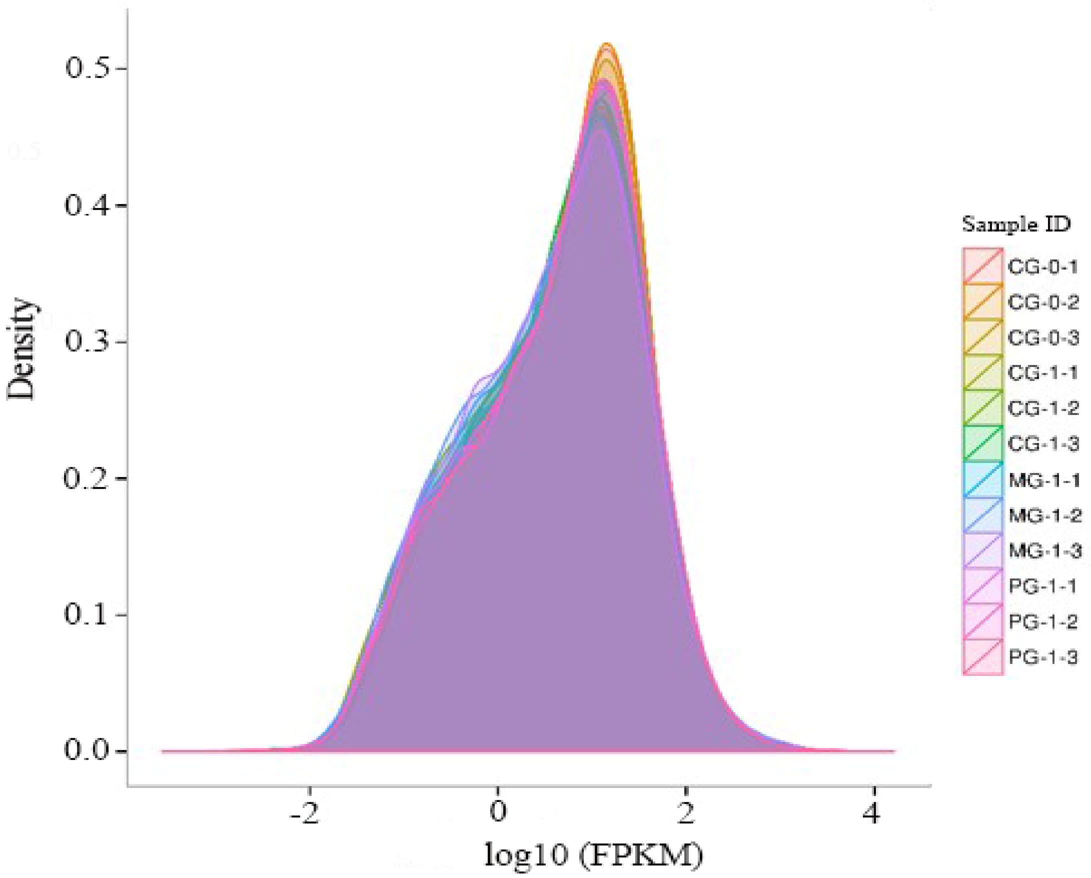

Figure S1. Comparison of FPKM density distribution of each sample.

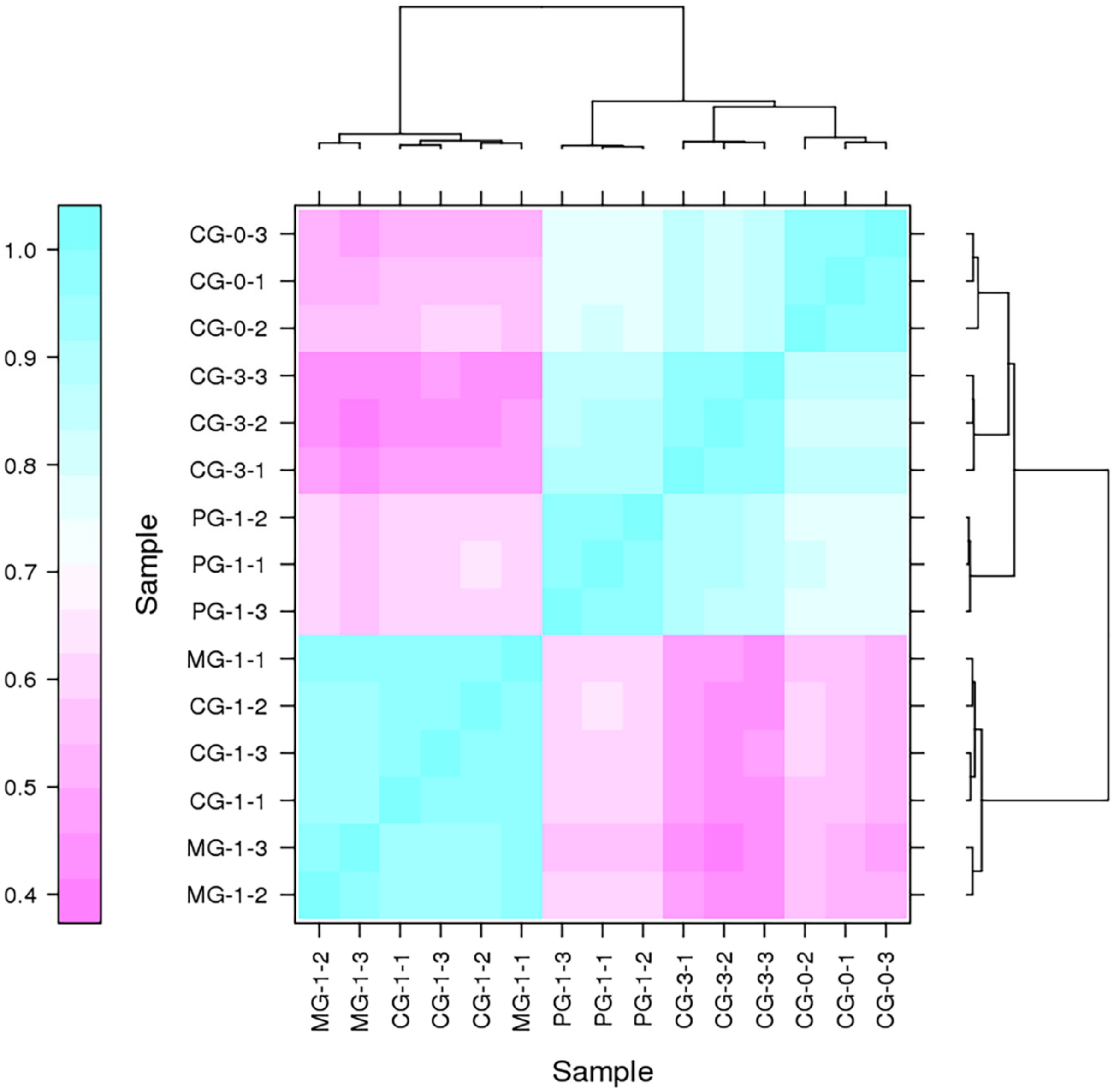

Figure **S2.** Heatmap of expression correlation among three groups.

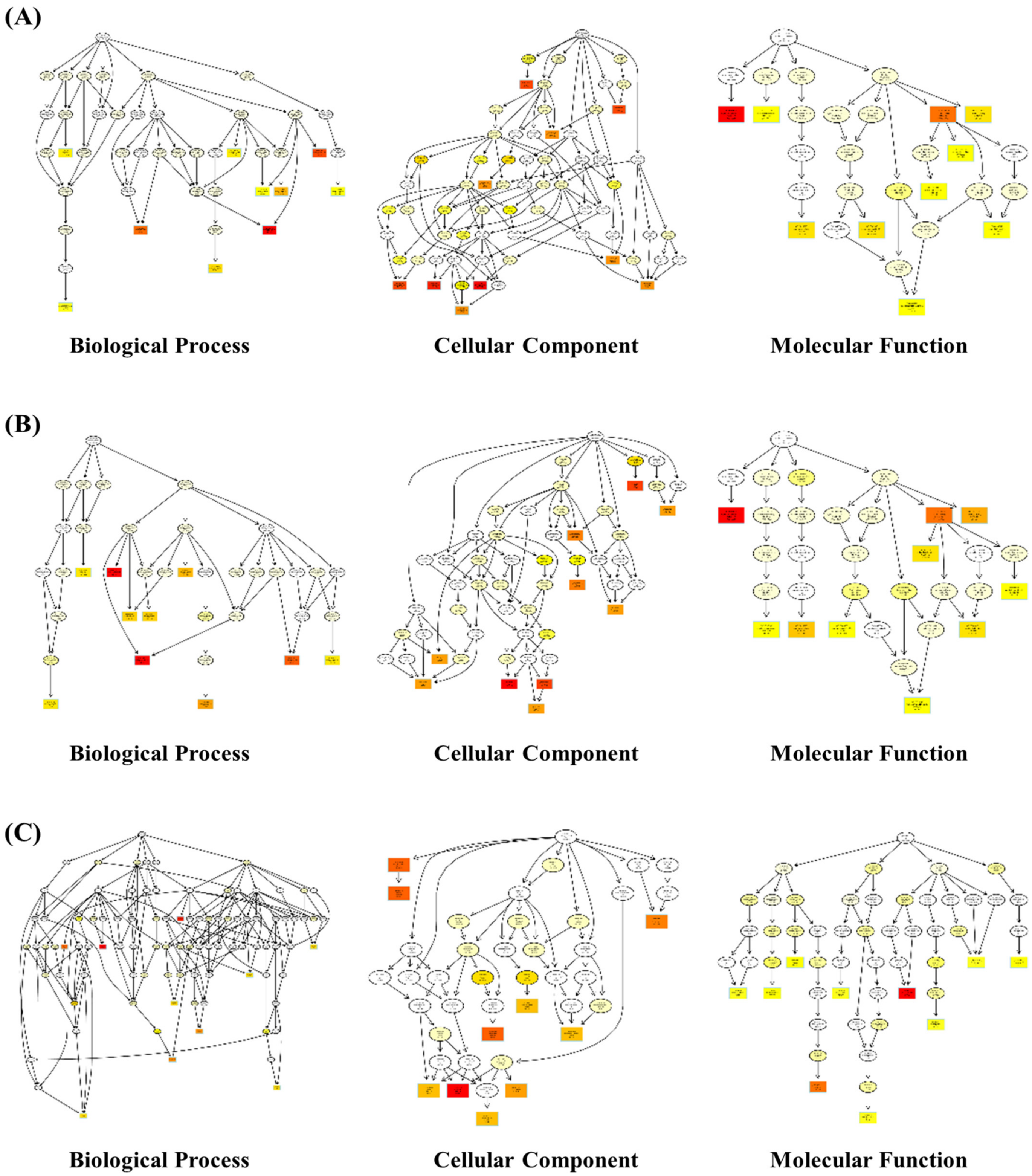

Figure S3. Functional enrichment analysis of DEGs. Directed Acyclic Graphs (DAGs) illustrating the hierarchical structure of significantly enriched GO terms in (**A**) CG-1 vs. CG-0, (**B**) MG-1 vs. CG-0, and (**C**) PG-1 vs. CG-0. The color depth (e.g., from yellow to red) indicates the significance level of enrichment, while the circles represent the GO terms.

(A)

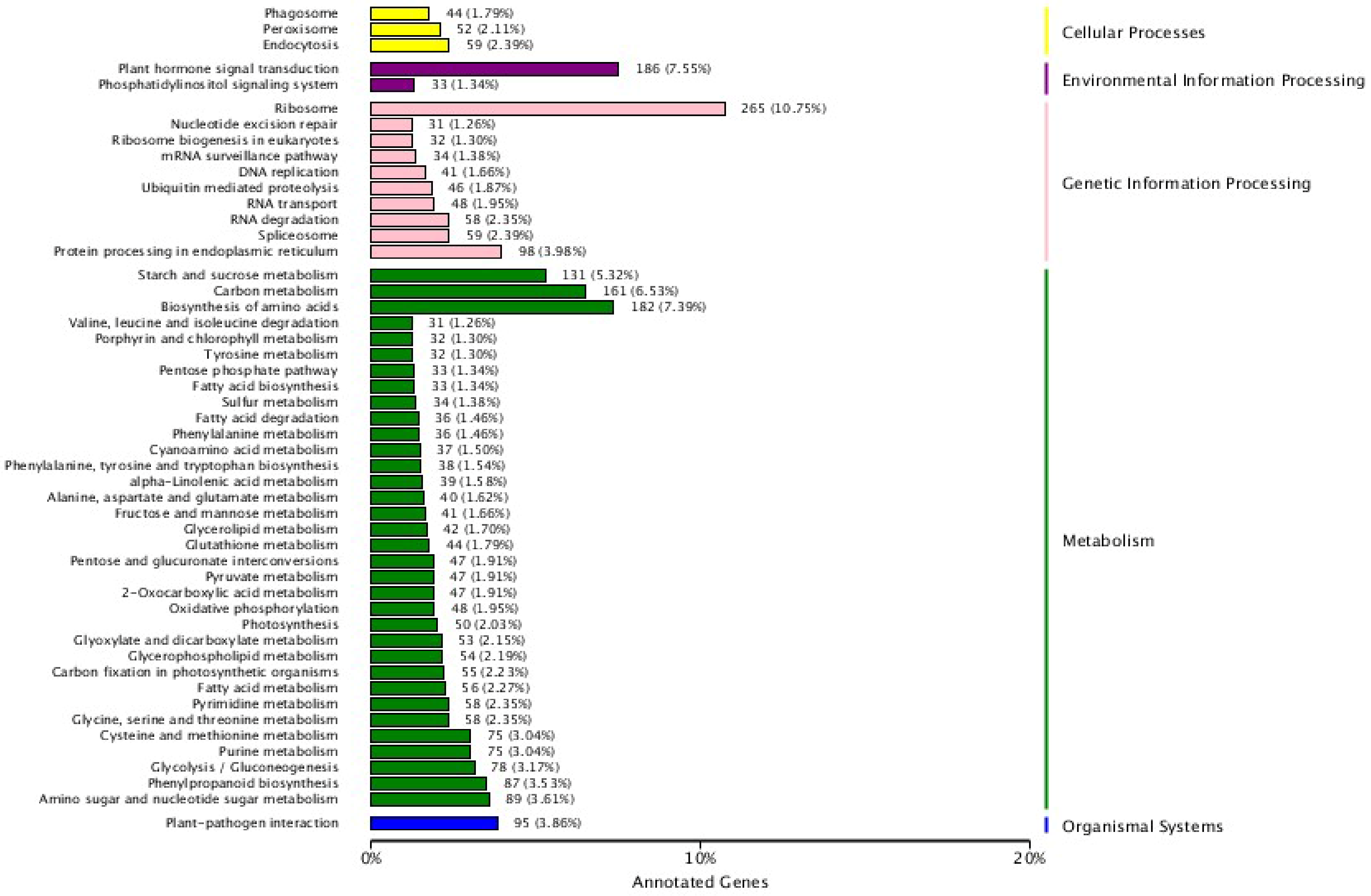

Phagosome
Peroxisome
Endocytosis
Plant hormone signal transduction
Phosphatidylinositol signaling system
Ribosome
Nucleotide excision repair
Ribosome biogenesis in eukaryotes
mRNA surveillance pathway
DNA replication
Ubiquitin mediated proteolysis
RNA transport
RNA degradation
Spliceosome
Protein processing in endoplasmic reticulum
Starch and sucrose metabolism
Carbon metabolism
Biosynthesis of amino acids
Valine, leucine and isoleucine degradation
Porphyrin and chlorophyll metabolism
Tyrosine metabolism
Pentose phosphate pathway
Fatty acid biosynthesis
Sulfur metabolism
Fatty acid degradation
Phenylalanine metabolism
Cyanoamino acid metabolism
Phenylalanine, tyrosine and tryptophan biosynthesis
alpha-Linolenic acid metabolism
Alanine, aspartate and glutamate metabolism
Fructose and mannose metabolism
Glycerolipid metabolism
Glutathione metabolism
Pentose and glucuronate interconversions
Pyruvate metabolism
2-Oxocarboxylic acid metabolism
Oxidative phosphorylation
Photosynthesis
Glyoxylate and dicarboxylate metabolism
Glycerophospholipid metabolism
Carbon fixation in photosynthetic organisms
Fatty acid metabolism
Pyrimidine metabolism
Glycine, serine and threonine metabolism
Cysteine and methionine metabolism
Purine metabolism
Glycolysis / Gluconeogenesis
Phenylpropanoid biosynthesis
Amino sugar and nucleotide sugar metabolism
Plant-pathogen interaction
44 (1.79%)
52 (2.11%)
59 (2.39%)
186 (7.55%)
33 (1.34%)
265 (10.75%)
31 (1.26%)
32 (1.30%)
34 (1.38%)
41 (1.66%)
46 (1.87%)
48 (1.95%)
58 (2.35%)
59 (2.39%)
98 (3.98%)
131 (5.32%)
161 (6.53%)
182 (7.39%)
31 (1.26%)
32 (1.30%)
32 (1.30%)
33 (1.34%)
33 (1.34%)
34 (1.38%)
36 (1.46%)
36 (1.46%)
37 (1.50%)
38 (1.54%)
39 (1.58%)
40 (1.62%)
41 (1.66%)
42 (1.70%)
44 (1.79%)
47 (1.91%)
47 (1.91%)
47 (1.91%)
48 (1.95%)
50 (2.03%)
53 (2.15%)
54 (2.19%)
55 (2.23%)
56 (2.27%)
58 (2.35%)
58 (2.35%)
75 (3.04%)
75 (3.04%)
78 (3.17%)
87 (3.53%)
89 (3.61%)
95 (3.86%)
Cellular Processes
Environmental Information Processing
Genetic Information Processing
Metabolism
Organismal Systems
0%
10%
20%
Annotated Genes

(B)

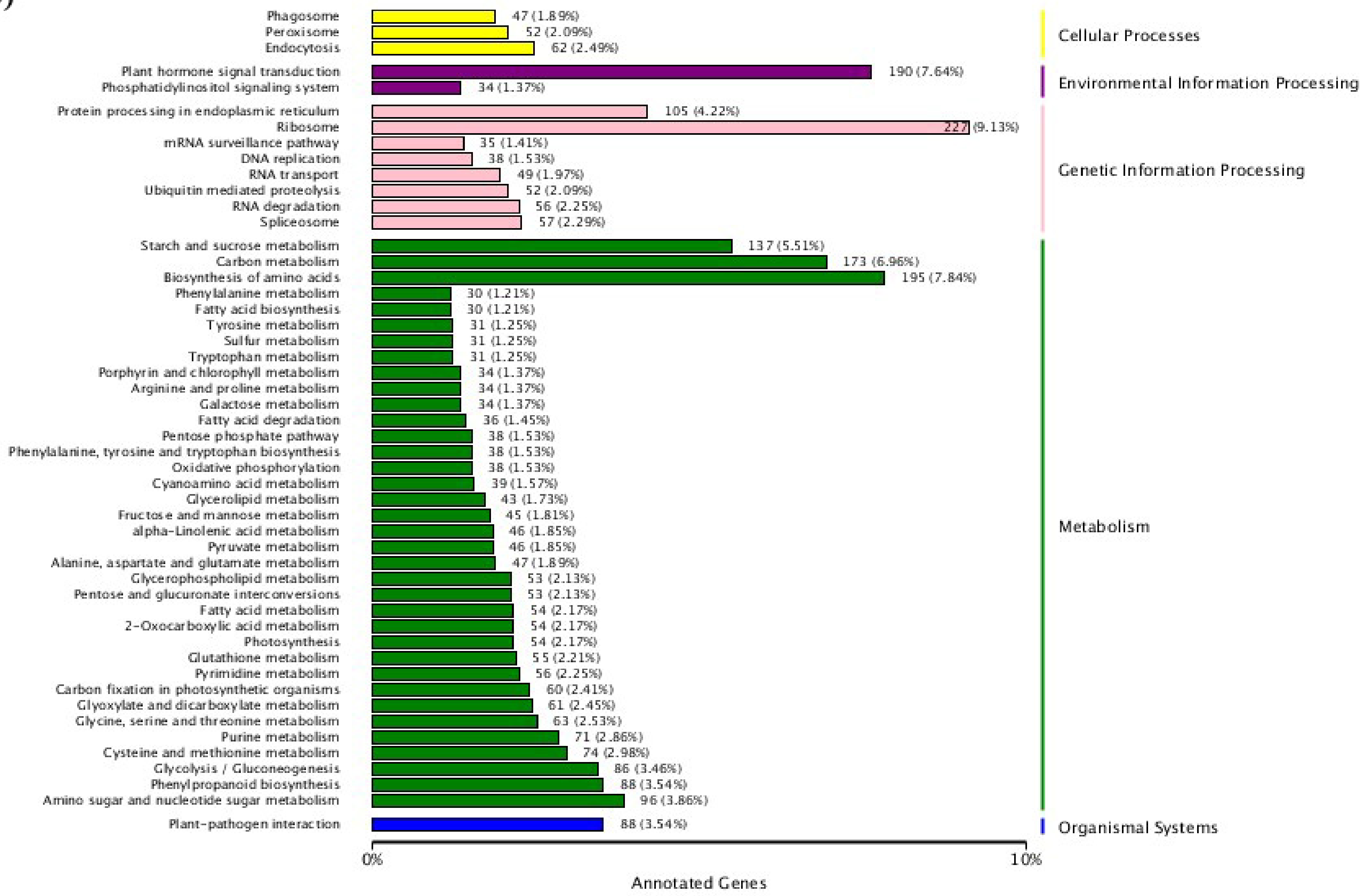

Phagosome
Peroxisome
Endocytosis
Plant hormone signal transduction
Phosphatidylinositol signaling system
Protein processing in endoplasmic reticulum
Ribosome
mRNA surveillance pathway
DNA replication
RNA transport
Ubiquitin mediated proteolysis
RNA degradation
Spliceosome
Starch and sucrose metabolism
Carbon metabolism
Biosynthesis of amino acids
Phenylalanine metabolism
Fatty acid biosynthesis
Tyrosine metabolism
Sulfur metabolism
Tryptophan metabolism
Porphyrin and chlorophyll metabolism
Arginine and proline metabolism
Galactose metabolism
Fatty acid degradation
Pentose phosphate pathway
Phenylalanine, tyrosine and tryptophan biosynthesis
Oxidative phosphorylation
Cyanoamino acid metabolism
Glycerolipid metabolism
Fructose and mannose metabolism
alpha-Linolenic acid metabolism
Pyruvate metabolism
Alanine, aspartate and glutamate metabolism
Glycerophospholipid metabolism
Pentose and glucuronate interconversions
Fatty acid metabolism
2-Oxocarboxylic acid metabolism
Photosynthesis
Glutathione metabolism
Pyrimidine metabolism
Carbon fixation in photosynthetic organisms
Glyoxylate and dicarboxylate metabolism
Glycine, serine and threonine metabolism
Purine metabolism
Cysteine and methionine metabolism
Glycolysis / Gluconeogenesis
Phenylpropanoid biosynthesis
Amino sugar and nucleotide sugar metabolism
Plant-pathogen interaction
47 (1.89%)
52 (2.09%)
62 (2.49%)
190 (7.64%)
34 (1.37%)
105 (4.22%)
227 (9.13%)
35 (1.41%)
38 (1.53%)
49 (1.97%)
52 (2.09%)
56 (2.25%)
57 (2.29%)
137 (5.51%)
173 (6.96%)
195 (7.84%)
30 (1.21%)
30 (1.21%)
31 (1.25%)
31 (1.25%)
31 (1.25%)
34 (1.37%)
34 (1.37%)
34 (1.37%)
36 (1.45%)
38 (1.53%)
38 (1.53%)
38 (1.53%)
39 (1.57%)
43 (1.73%)
45 (1.81%)
46 (1.85%)
46 (1.85%)
47 (1.89%)
53 (2.13%)
53 (2.13%)
54 (2.17%)
54 (2.17%)
54 (2.17%)
55 (2.21%)
56 (2.25%)
60 (2.41%)
61 (2.45%)
63 (2.53%)
71 (2.86%)
74 (2.98%)
86 (3.46%)
88 (3.54%)
96 (3.86%)
88 (3.54%)
Cellular Processes
Environmental Information Processing
Genetic Information Processing
Metabolism
Organismal Systems
0%
10%
Annotated Genes

(C)

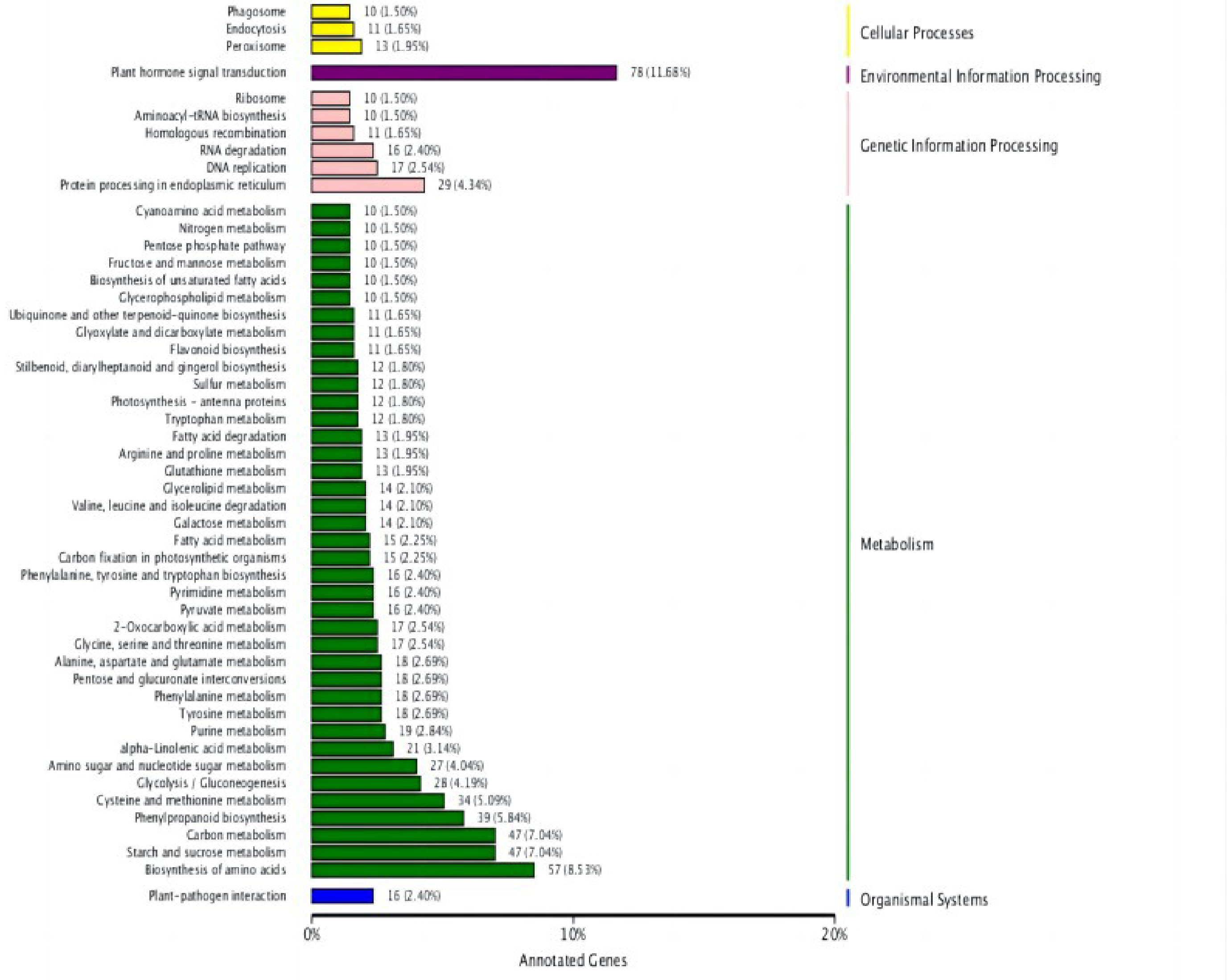

Figure S4. KEGG pathway classification of DEGs in three comparisons. (**A**) KEGG classification for CG-1 vs CG-0. (**B**) KEGG classification for MG-1 vs CG-0. (**C**) KEGG classification for PG-1 vs CG-0. The x-axis indicates the percentage (or number) of genes, and the y-axis lists the KEGG sub-categories classified into five major branches (Metabolism, Genetic Information Processing, Environmental Information Processing, Cellular Processes, and Organismal Systems).

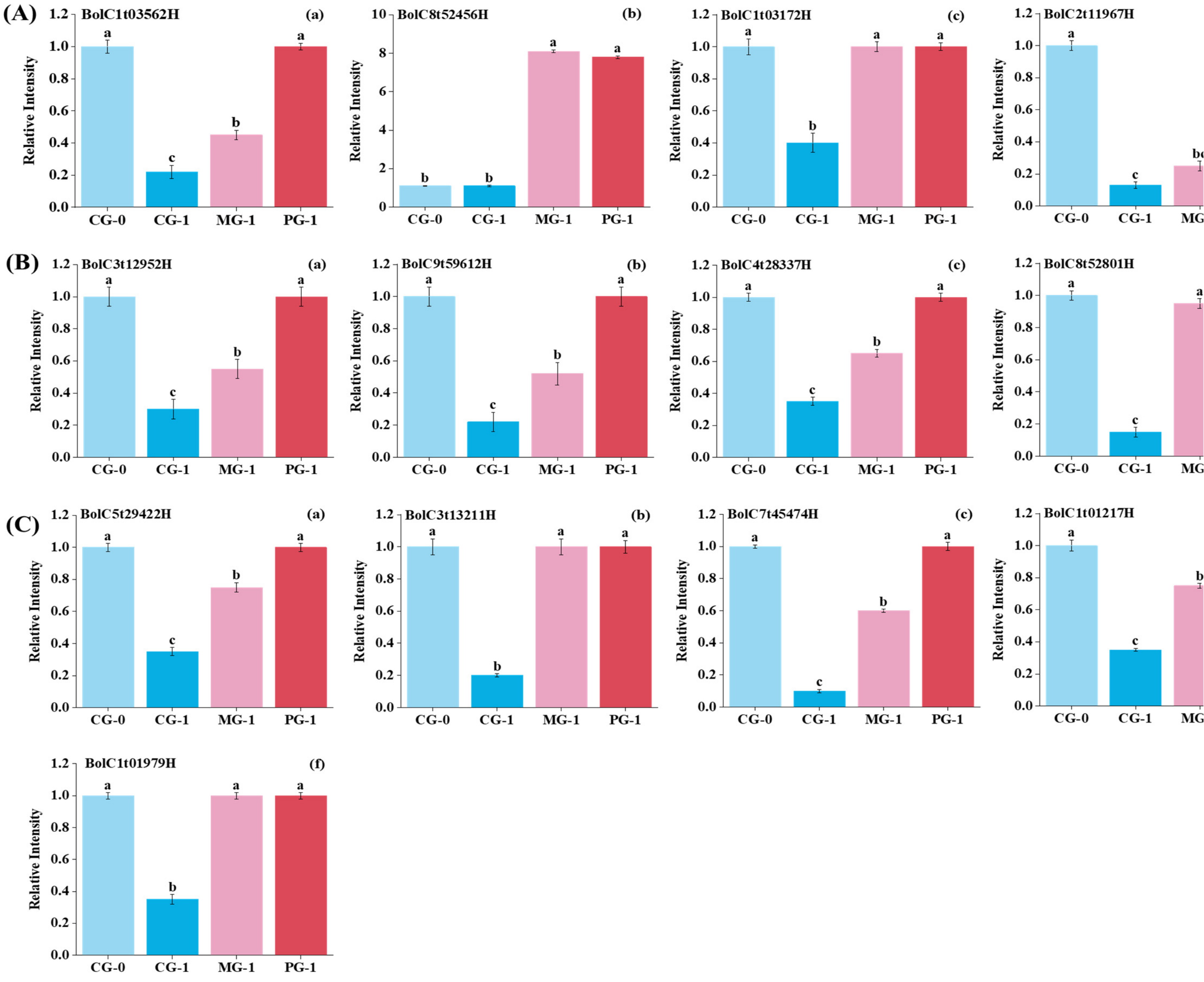
(A)
BolC1t03562H
(a)
BolC8t52456H
(b)
BolC1t03172H
(c)
BolC2t11967H
(B)
BolC3t12952H
(a)
BolC9t59612H
(b)
BolC4t28337H
(c)
BolC8t52801H
(C)
BolC5t29422H
(a)
BolC3t13211H
(b)
BolC7t45474H
(c)
BolC1t01217H
BolC1t01979H
(f)
Relative Intensity
CG-0
CG-1
MG-1
PG-1

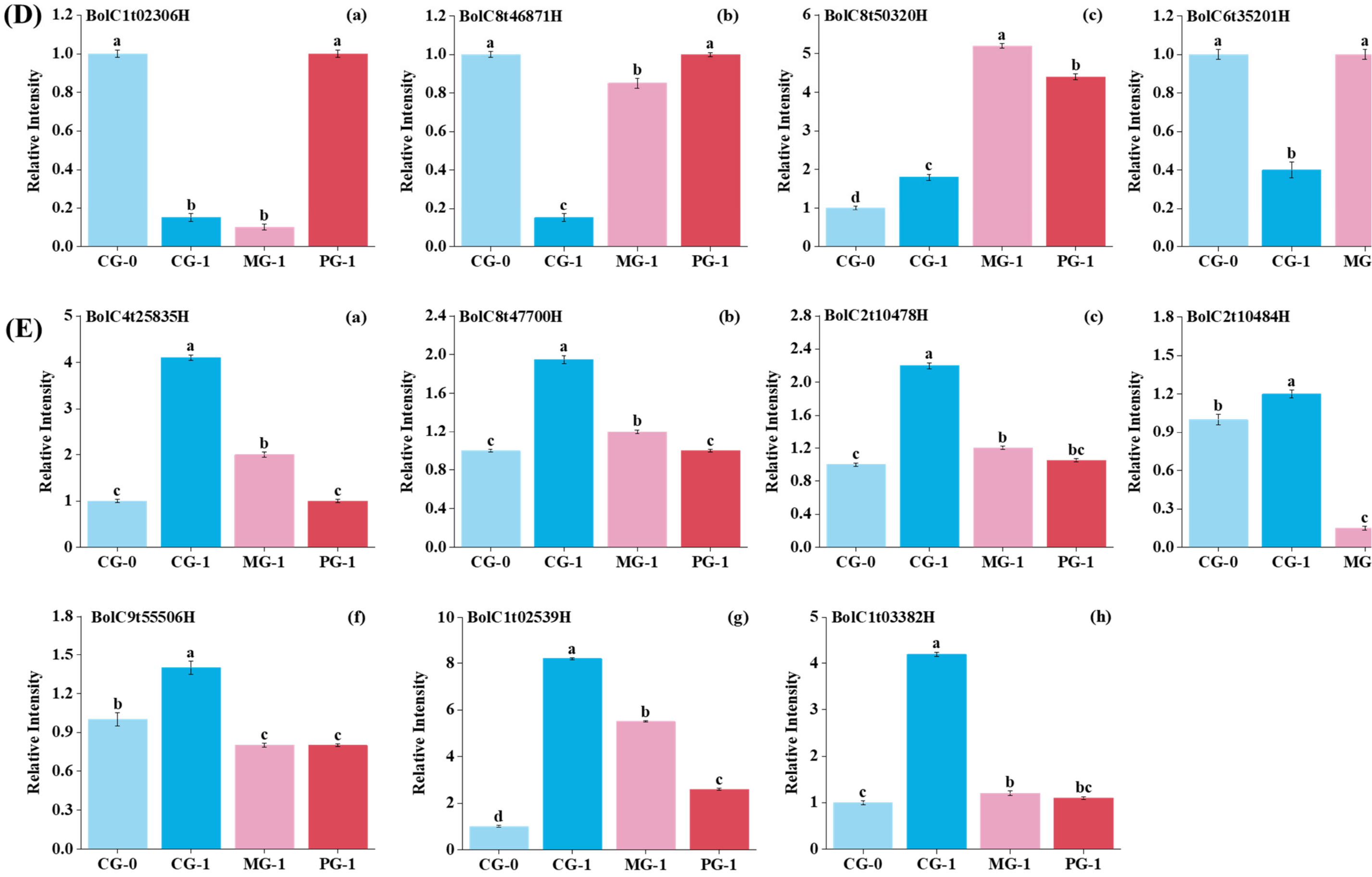

Figure S5. Detailed relative expression profiles of all identified key genes involved in chlorophyll metabolism. (**A**) GluTR genes; (**B**) PBGD (a, b), UROD (c), and CPOX (d) genes; (**C**) MgCh (a, b, c, d) and MgMT (e, f) genes; (**D**) POR (a, b), CS (c), and CAO (d, e) genes; (**E**) CBR (a, b), CLH (c, d, e, f), PPH (g), and PaO (h) genes.
